# Cucurbitane-Type Triterpene Glycosides from *Momordica charantia* and Their *α*-Glucosidase Inhibitory Activities

**DOI:** 10.1007/s13659-020-00241-5

**Published:** 2020-05-06

**Authors:** Ya Gao, Jian-Chao Chen, Xing-Rong Peng, Zhong-Rong Li, Hai-Guo Su, Ming-Hua Qiu

**Affiliations:** 1 State Key Laboratory of Phytochemistry and Plant Resources in West China, Kunming Institute of Botany, Chinese Academy of Sciences, Kunming, 650201 People’s Republic of China; 2 University of the Chinese Academy of Sciences, Beijing, 100049 People’s Republic of China; 3 Yunnan Key Laboratory of Natural Medicinal Chemistry, Chinese Academy of Sciences, Kunming, 650201 People’s Republic of China

**Keywords:** *Momordica charantia*, Cucurbitane-type triterpene glycosides, *α*-Glucosidase inhibitory activity

## Abstract

**Abstract:**

Ten cucurbitane-type triterpene glycosides, including five new compounds named charantosides H (**1**), J (**2**), K (**3**), momorcharacoside A (**4**), goyaglycoside-l (**5**), and five known compounds (**6–10**), were isolated from the EtOAc extract of *Momordica charantia* fruits. The chemical structures of these compounds were identified by 1D and 2D NMR and HRESIMS spectroscopic analyses. Configurations of new compounds were determined by ROESY correlations and comparison of their ^13^C NMR data with literature reported values. All compounds were evaluated for their inhibition against *α*-glucosidase, in which compounds **2**, **5**, **7**, **8**, **9** showed moderate inhibitory activities with IC_50_ values ranging from 28.40 to 63.26 μM comparing with the positive control (acarbose, IC_50_ 87.65 ± 6.51 μM).

**Graphic Abstract:**

**Electronic supplementary material:**

The online version of this article (10.1007/s13659-020-00241-5) contains supplementary material, which is available to authorized users.

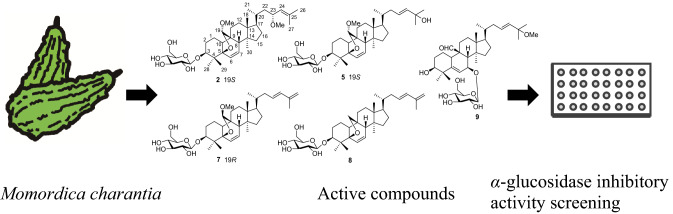

## Introduction

Diabetes mellitus (DM) is a type of metabolic disorder caused by insufficient insulin secretion or insulin utilization disorder, and is marked by persistent hyperglycemia [1]. The latest edition of the International Diabetes Federation (IDF)ʼs Diabetes Atlas estimates that, in 2019, about 463 million adults were living with DM around the world, and 11.3% of global deaths were due to DM [2]. Moreover, it can overwhelm the social and economic welfare of all countries, regardless of their economic level. Therefore, the prevention and treatment of DM is essential. In modern medicine, there are many oral hypoglycemic agents (OHAs) used to treat DM, however, they also have different adverse effects including gastrointestinal upset, lactic acidosis, characteristic hepatocyte injury, dizziness, acute hypoglycemia, and even death [3, 4]. Therefore, screening the new hypoglycemic drugs with high efficiency and low toxicity from the natural products of plants is urgently required. In China, ancient books have recorded many Chinese herbs used in the treatment of DM, among which *Momordica charantia* (Cucurbitaceae) was very popular and had an incredible hypoglycemic effect. Therefore, *M. charantia* has great research potential in reducing blood sugar and is a hot spot in modern phytochemical research.

*Momordica charantia* is a traditional medicinal and edible plant, which has a long history of use in developing countries. Modern phytochemistry research shows that both crude extracts and secondary metabolites (including polysaccharides, triterpenes, saponins, proteins, flavonoids, alkaloids, and steroids, etc.) of *M. charantia* possess anti-diabetic activity [5–10]. Thereinto, cucurbitane-type triterpenoids are the main bioactive ingredients in *M. charantia*, which can control blood sugar through multiple mechanisms of action (such as PPAR-*γ* activator, PTP1B inhibitor, and *α*-glucosidase inhibitor, etc.) [11, 12]. To date, more than 300 kinds of cucurbitane-type triterpenoids have been identified, and some of them showed prominent biological activity [6, 10, 13]. Based on this, we conducted further excavations, hoping to find new cucurbitane-type triterpenoids with good hypoglycemic activity.

## Results, Discussion and Conclusion

**Fig. 1:**
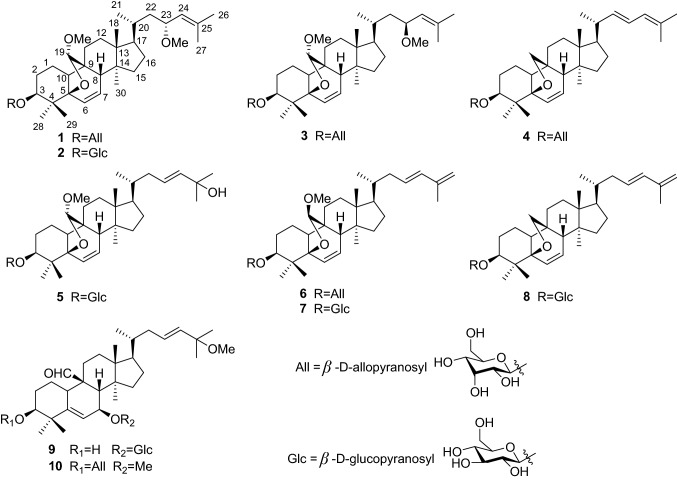
Chemical structures of compounds **1–10** from *M. charantia*

Phytochemical investigation of the fruits of *M. charantia* resulted in the isolation of five new compounds, charantosides H (**1**), J (**2**), K (**3**), momorcharacoside A (**4**), and goyaglycoside-l (**5**), by repeated column chromatography (CC) (Fig. 1). Meanwhile, five known compounds (**6–10**) were isolated and identified, on the basis of comparison of obtained values with literature values, as (19*R*,23*E*)-5*β*,19-epoxy-19-methoxycucurbita-6,23,25-trien-3*β*-ol 3-*O*-*β*-d-allopyranoside (**6**) [14], charantoside I (**7**) [15], charantoside III (**8**) [15], momordicoside K (**9**) [16], and 7*β*,25-dimethoxycucurbita-5(6),23(*E*)-dien-19-al 3-*O*-*β*-d-allopyranoside (**10**) [17] (Fig. 1), respectively.

**Fig. 2:**
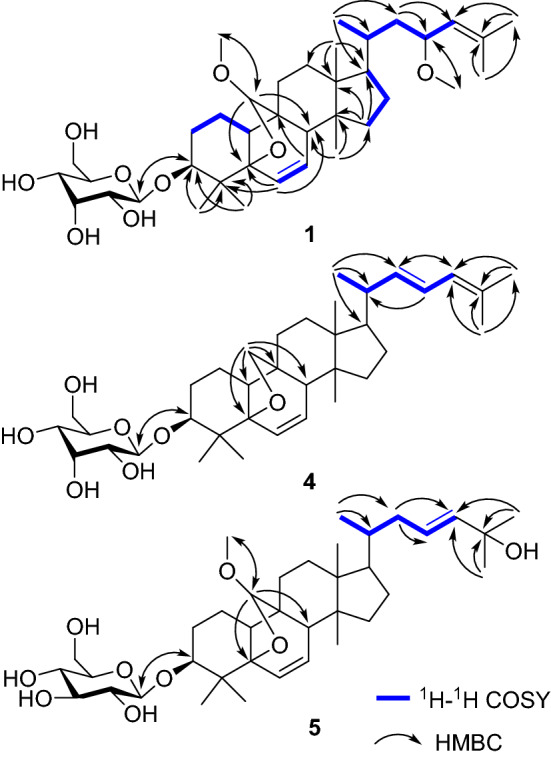
Key ^1^H-^1^H COSY and HMBC correlations of **1**, **4**, and **5**

**Fig. 3:**
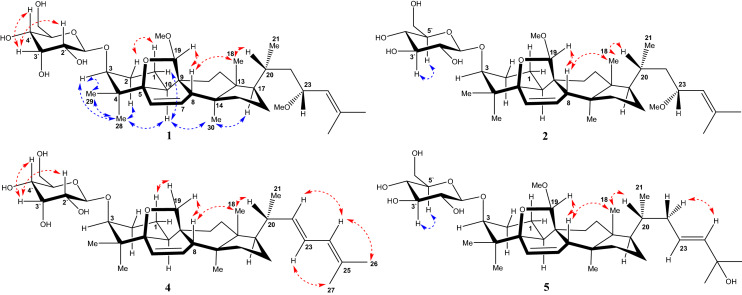
Key ROESY correlations of **1**, **2**, **4**, and **5**

All of the five new compounds were considered as monoglycosides based on the IR absorption bands of a glycosidic function (e.g., **1**: *ν*_max_ 3426, 1084, 1033 cm^−1^) [15, 18] and an anomeric proton signal of the glycosyl moiety observed in their ^1^H NMR spectra. After acid hydrolysis, the sugars of **4** and **5** were identified as d-allose and d-glucose by comparing their TLC and specific rotation with the corresponding authentic sample. The predicted structures for these new compounds as depicted below were supported by analysis of the ^1^H-^1^H COSY, HMBC, and NOESY data (Figs. 2 and 3), in addition to ^13^C-DEPT, and HMQC data.

Compound **1** was obtained as white amorphous powder. The molecular formula C_38_H_62_O_9_ was deduced from its HRESIMS (positive-ion mode) data (685.4295 [M + Na]^+^), indicated eight degrees of unsaturation. The ^1^H NMR spectrum of **1** (Table 1) showed signals assignable to seven methyl groups [*δ*_H_ 1.73, 1.69, 1.47, 1.05, 0.88, 0.86, 0.84], two methoxy groups [*δ*_H_ 3.58, 3.29], three olefinic protons [*δ*_H_ 6.27 (1H, dd, *J* = 1.8, 9.0 Hz), 5.48 (1H, dd, *J* = 3.6, 9.6 Hz), 5.22 (1H, d, *J* = 8.4 Hz)], and an anomeric proton [*δ*_H_ 5.36 (1H, d, *J* = 7.8 Hz, H-1′)]. The ^13^C NMR (Table 2) showed 38 carbon signals. The DEPT spectrum exhibited nine methyls, eight methylenes, fifteen methines, and six quaternary carbons. And ^13^C NMR spectrum showed olefinic carbons appeared at *δ*_C_ 135.1, 134.4, 129.1, and 127.7. The NMR data of **1** were closely similar to those of (19*R*,23*R*)-5*β*,19-epoxy-19,23-dimethoxycucurbita-6,24-dien-3*β*-ol 3-*O*-*β*-d-allopyranoside (charantoside II) [15] except for the signals due to the stereochemistry at C-19. And that Δ*δ*_C_ values [*δ*_C_ (charantoside II) − *δ*_C_ (**1**)] for the relevant signals were calculated as 1.7 (C-5), − 7.3 (C-8), − 0.7 (C- 9), 2.8 (C-10), 1.6 (C-11) and -1.4 (C-19) from the ^13^C NMR data of charantoside II and **1**, which were feckly consistent with the Δ*δ*_C_ values [*δ*_C_ (19*R*) − *δ*_C_ (19*S*)] of 1.8 (C-5), − 7.8 (C-8), − 0.7 (C-9), 2.6 (C-10), 1.8 (C-11) and -2.6 (C-19) calculated from the ^13^C NMR data of 5*β*,(19*R*)- and 5*β*,(19*S*)-epoxy-19,23-dimethoxycucurbita-6,24-dien-3β-ol [19]. Therefore, compound **1** has the (*S*)-configuration at C-19, and the ROESY correlation (Fig. 3) of H-8/H-19 confirmed the above deduction [20]. The actual connection positions were further established on the basis of HMBC correlations (Fig. 2) between H-1′ (*δ*_H_ 5.36) of the sugar moiety and C-3 (*δ*_C_ 84.7) of the aglycon group. And we found that the NMR data of the sugar moiety of **1** was basically consistent with **4**, which corroborates the presence of a d-allose. In addition, long-range correlations were also observed at 19-methoxyl protons (*δ*_H_ 3.58)/C-19 (*δ*_C_ 113.8), and 23-methoxyl protons (*δ*_H_ 3.29)/C-23 (*δ*_C_ 74.6). Therefore, the molecular formula of **1**, along with the 1D and 2D spectroscopic data illustrated that the structure of **1** could be assigned as (19*S*,23*R*)-5*β*,19-epoxy-19,23-dimethoxycucurbita-6,24-dien-3*β*-ol 3-*O*-*β*-d-allopyranoside, named charantoside H.

**Table 1:** ^1^H NMR spectroscopic data of compounds **1**–**5** in pyridine-d5 [δ in ppm, J in Hz]

No.	**1**^a^	**2**^a^	**3**^b^	**4**^a^	**5**^a^
1	1.27 (1H, overlap) 2.49 (1H, m)	1.35 (1H, overlap) 2.48 (1H, overlap)	1.33 (1H, m) 2.51 (1H, m)	1.28 (1H, overlap) 1.34 (1H, overlap)	1.26 (1H, overlap) 2.48 (1H, m)
2	1.75 (1H, overlap) 2.28 (1H, overlap)	1.25 (1H, d, 0.8) 2.33 (1H, m)	1.74 (1H, m) 2.27 (1H, m)	1.75 (1H, overlap) 2.36 (1H, d, 9.0)	1.77 (1H, overlap) 2.31 (1H, overlap)
3	3.63 (1H, s)	3.65 (1H, t, 2.8)	3.63 (1H, t, 2.4)	3.63 (1H, s)	3.65 (1H, s)
6	6.27 (1H, dd, 1.8, 9.0)	6.28 (1H, d, 10.8)	6.27 (1H, dd, 1.6, 9.6)	6.17 (1H, dd, 1.8, 10.2)	6.28 (1H, d, 9.6)
7	5.48 (1H, dd, 3.6, 9.6)	5.46 (1H, dd, 3.6, 9.6)	5.48 (1H, dd, 3.2, 9.6)	5.53 (1H, dd, 3.6, 9.6)	5.48 (1H, dd, 3.6, 9.6)
8	2.28 (1H, s)	2.28 (1H, m)	2.30 (1H, m)	2.28 (1H, s)	2.29 (1H, s)
10	2.32 (1H, dd, 5.4, 12.6)	2.32 (1H, m)	2.33 (1H, dd, 5.6, 12.8)	2.26 (1H, m)	2.28 (1H, overlap)
11	1.78 (1H, m) 1.87 (1H, m)	1.78 (1H, overlap) 1.87 (1H, overlap)	1.81 (1H, m) 1.89 (1H, m)	1.32 (1H, overlap) 1.60 (1H, m)	1.77 (1H, m) 1.83 (1H, m)
12	1.60 (1H, m) 1.62 (1H, m)	1.45 (1H, overlap) 1.60 (1H, overlap)	1.60 (1H, m) 1.79 (1H, overlap)	1.41 (1H, overlap) 1.55 (1H, m)	1.39 (1H, overlap) 1.48 (1H, overlap)
15	1.19 (1H, m) 1.26 (1H, m)	1.18 (1H, m) 1.27 (1H, m)	1.22 (1H, m) 1.28 (1H, m)	1.19 (1H, overlap) 1.34 (1H, overlap)	1.21 (1H, m) 1.27 (1H, overlap)
16	1.41 (1H, overlap) 1.96 (1H, overlap)	1.45 (1H, overlap) 1.98 (1H, overlap)	1.35 (1H, m) 1.96 (1H, m)	1.18 (1H, m) 1.31 (1H, m)	1.12 (1H, m) 1.91 (1H, m)
17	1.44 (1H, m)	1.44 (1H, m)	1.53 (1H, m)	1.51 (1H, m)	1.49 (1H, overlap)
18	0.84 (3H, s)	0.83 (3H, s)	0.83 (3H, s)	0.77 (3H, s)	0.79 (3H, s)
19	4.61 (1H, s)	4.59 (1H, s)	4.61 (1H, s)	3.59 (1H, d, 7.8) 3.76 (1H, d, 8.4)	4.62 (1H, s)
20	1.93 (1H, m)	1.94 (1H, m)	1.55 (1H, m)	2.16 (1H, m)	1.49 (1H, overlap)
21	1.05 (3H, s)	1.03 (3H, d, 6.4)	1.03 (3H, d, 5.6)	1.04 (3H, d, 6.6)	0.92 (3H, d, 5.4)
22	1.04 (1H, s) 1.85 (1H, overlap)	1.03 (1H, overlap) 1.84 (1H, m)	1.58 (1H, m) 1.70 (1H, overlap)	5.48 (1H, dd, 8.4, 14.4)	1.84 (1H, m) 2.24 (1H, dd, 4.2, 12.0)
23	4.13 (1H, td, 3.0, 10.2)	4.13 (1H, m)	4.10 (1H, m)	6.33 (1H, dd, 10.8, 15.0)	5.92 (1H, overlap)
24	5.22 (1H, d, 8.4)	5.20 (1H, d, 8.8)	5.15 (1H, dt, 1.6, 9.6)	5.91 (1H, d, 10.2)	5.92 (1H, overlap)
26	1.73 (3H, s)	1.72 (3H, s)	1.73 (3H, s)	1.72 (3H, s)	1.54 (3H, overlap)
27	1.69 (3H, s)	1.68 (3H, s)	1.71 (3H, s)	1.73 (3H, s)	1.54 (3H, overlap)
28	0.86 (3H, s)	0.90 (3H, s)	0.86 (3H, s)	0.87 (3H, s)	0.89 (3H, s)
29	1.47 (3H, s)	1.54 (3H, s)	1.47 (3H, s)	1.46 (3H, s)	1.54 (3H, s)
30	0.88 (3H, s)	0.88 (3H, s)	0.88 (3H, s)	0.85 (3H, s)	0.82 (3H, s)
19-OCH_3_	3.58 (3H, s)	3.43 (3H, s)	3.55 (3H, s)		3.44 (3H, s)
23-OCH_3_	3.29 (3H, s)	3.28 (3H, s)	3.29 (3H, s)		
1′	5.36 (1H, d, 7.8)	4.89 (1H, d, 7.2)	5.37 (1H, d, 8.0)	5.38 (1H, d, 7.8)	4.92 (1H, d, 7.8)
2′	3.93 (1H, t, 3.6)	4.03 (1H, t, 8.0)	3.93 (1H, d, 8.0)	3.95 (1H, dd, 2.4, 7.8)	4.03 (1H, t, 8.4)
3′	4.71 (1H, s)	3.96 (1H, m)	4.70 (1H, t, 2.4)	4.69 (1H, t, 2.4)	3.96 (1H, d, 4.2)
4′	4.23 (1H, m)	4.25 (1H, m)	4.25 (1H, d, 8.8)	4.16 (1H, dd,3.0, 9.6)	4.26 (1H, overlap)
5'	4.46 (1H, m)	4.26 (1H, overlap)	4.46 (1H, m)	4.46 (1H, m)	4.25 (1H, overlap)
6'	4.40 (1H, m) 4.53 (1H, d, 4.4)	4.42 (1H, dd, 4.8, 11.6) 4.56 (1H, d, 1.6)	4.41 (1H, dd, 4.8, 11.2) 4.53 (1H, dd, 1.6, 11.2)	4.38 (1H, dd, 5.4, 11.4) 4.52 (1H, dd, 1.8, 12.0)	4.42 (1H, dd, 4.8, 11.4) 4.57 (1H, dd, 1.8, 12.0)

^a^Recorded at 600 MHz in pyridine-d_5_

^b^Recorded at 800 MHz in pyridine-*d*_5_

**Table 2:** ^13^C (150 MHz) NMR spectroscopic data of compounds **1**–**5** in pyridine-d5 [δ in ppm]

No.	**1**	**2**	**3**	**4**	**5**
1	18.1 t	18.0 t	18.1 t	18.8 t	18.1 t
2	27.4 t	27.3 t	27.5 t	27.4 t	27.4 t
3	84.7 d	85.0 d	84.7 d	85.2 d	85.0 d
4	38.7 s	38.8 s	38.8 s	38.8 s	38.9 s
5	83.8 s	83.8 s	83.8 s	85.8 s	83.8 s
6	135.1 d	135.1 d	135.2 d	133.9 d	135.2 d
7	129.1 d	129.1 d	129.1 d	129.8 d	129.1 d
8	49.5 d	49.5 d	49.6 d	52.1 d	49.6 d
9	48.9 s	48.9 s	49.0 s	45.1 s	49.0 s
10	38.8 d	38.9 d	38.9 d	40.0 d	38.9 d
11	21.8 t	21.8 t	21.8 t	23.7 t	21.8 t
12	30.8 t	30.8 t	30.8 t	30.9 t	30.6 t
13	45.3 s	45.3 s	45.3 s	45.3 s	45.2 s
14	48.1 s	48.1 s	48.2 s	48.8 s	48.2 s
15	33.5 t	33.5 t	33.6 t	33.2 t	33.6 t
16	28.1 t	28.1 t	28.4 t	28.6 t	27.9 t
17	51.2 d	51.2 d	51.3 d	50.4 d	50.2 d
18	14.9 q	14.9 q	14.8 q	15.1 q	15.0 q
19	113.8 d	113.6 d	113.7 d	79.9 t	113.6 d
20	32.6 d	32.6 d	33.7 d	40.5 d	36.5 d
21	18.8 q	18.8 q	19.8 q	20.6 q	18.8 q
22	43.2 t	43.2 t	42.9 t	138.9 d	39.4 t
23	74.6 d	74.6 d	76.3 d	124.6 d	123.6 d
24	127.7 d	127.7 d	127.2 d	126.1 d	141.6 d
25	134.4 s	134.4 s	136.0 s	132.1 s	69.6 s
26	25.7 q	25.7 q	25.7 q	25.7 q	30.8 q
27	18.0 q	18.0 q	18.3 q	18.1 q	30.8 q
28	25.0 q	25.1 q	25.1 q	25.4 q	25.1 q
29	20.8 q	20.9 q	20.9 q	20.8 q	21.0 q
30	20.0 q	19.9 q	20.0 q	20.1 q	20.0 q
19-OCH3	55.7 q	55.5 q	55.6 q		55.4 q
23-OCH3	55.4 q	55.4 q	55.2 q		
Sugar moiety	All	Glc	All	All	Glc
1′	103.7 d	106.5 d	103.7 d	104.0 d	106.5 d
2′	73.1 d	75.8 d	73.2 d	72.8 d	76.0 d
3′	72.3 d	78.2 d	72.3 d	72.4 d	78.3 d
4′	68.9 d	71.6 d	69.0 d	69.1 d	71.7 d
5′	75.7 d	78.3 d	75.8 d	75.9 d	78.5 d
6′	63.0 t	62.8 t	63.1 t	63.1 t	62.9 t

Compound **2** was obtained as white solid. It showed a quasi-molecular ion at 685.4293([M + Na]^+^) in the HRESIMS (positive-ion mode) spectrum and had the same molecular formula C_38_H_62_O_9_ as **1**, which also possessed eight degrees of unsaturation. Detailed analysis of the ^1^H, ^13^C NMR, and DEPT spectra (Tables 1 and 2) of compound **2**, which showed heavily resemblance in all signals to those of (19*S*,23*R*)-5*β*,19-epoxy-19,23-dimethoxycucurbita-6,24-dien-3*β*-ol 3-*O*-*β*-d-allopyranoside (charantoside H, **1**) except that d-allose of **1** were replaced by d-glucose in **2**. In the ^13^C NMR spectrum of compound **2**, an anomeric carbon atom (*δ*_C_ 105.2) and a series of oxygenated carbon signals (*δ*_C_ 78.6, 77.8, 76.1, 71.8, and 62.9) were in line with **5**, confirmed the presence of a *β*-d-glucopyranosyl residue [15]. The ^1^H-^1^H COSY and HMBC correlations of compound **1** and **2** were similar, but their ROESY spectra (Fig. 3) showed the different correlations between H-3′/H-5′, further proved the type of sugar moiety of **2**. Similarly, the absolute configuration of C-19 (*S*) in **2** was confirmed by ROESY correlation of H-8/H-19. Based on the above corroboration, the structure of compound **2** was identified as (19*S*,23*R*)-5*β*,19-epoxy-19,23-dimethoxycucurbita-6,24-dien-3*β*-ol 3-*O*-*β*-d-glucopyranoside, named charantoside J.

Compound **3** was obtained as white amorphous powder. It revealed a quasi-molecular ion at 685.4296 ([M + Na]^+^) in the HRESIMS (positive-ion mode) spectrum and had the same molecular formula C_38_H_62_O_9_ as **1**. According to the ^1^H, ^13^C NMR, and DEPT spectra (Tables 1 and 2) of **3**, which were also similar to those of charantoside H (**1**) except for the signals due to the stereochemistry at C-23. Compound **3** exhibited ^1^H NMR signals (Table 1) for the side-chain protons at *δ* 1.03 (3H, d, *J* = 5.6 Hz, a secondary methyl), 1.69 and 1.73 (each 3H, s, two vinylic methyls), 3.29 (3H, s, an *O*-methyl), 4.10 (1H, m, an allylic oxymethine), and 5.15 (1H, dt, *J* = 1.6, 9.6 Hz, an olefinic methine). Detailed comparisons of its ^13^C NMR data (Table 2) with those of compound **1**, the Δ*δ*_C_ values [Δ*δ*_C_ (**1**) − Δ*δ*_C_ (**3**)] for the side-chain signals were calculated as − 1.1 (C-20), − 1.0 (C-21), + 0.3 (C-22), − 1.7 (C-23), + 0.5 (C-24), − 1.6 (C-25), − 0.0 (C-26), and − 0.3 (C-27), which were almost in line with the Δ*δ*_C_ values [Δ*δ*_C_ (23*R*) − Δ*δ*_C_ (23*S*)] of − 0.9 (C-20), − 0.9 (C-21), + 0.4 (C-22), − 1.6 (C-23), + 0.5 (C-24), − 1.4 (C-25), − 0.1 (C-26), and − 0.4 (C-27) calculated from the ^13^C NMR data of charantoside II (23*R*) and charantoside VI (23*S*) [15]. As a consequence, compound **3** has the (*S*)-configuration at C-23. The ^1^H-^1^H COSY, HMBC, and ROESY correlations of compounds **1** and **3** were similar as well, suggesting that both compounds **1** and **3** have an almost identical planar chemical structure. Analogously, ROESY correlation of H-8/H-19 certified that acetal carbon (C-19) should has the (*S*)-configuration. Eventually, the structure of compound **3** was identified as (19*S*,23*S*)-5*β*,19-epoxy-19,23-dimethoxycucurbita-6,24-dien-3*β*-ol 3-*O*-*β*-d-allopyranoside, named charantoside K.

Compound **4** was obtained as white amorphous powder and assigned a molecular formula of C_36_H_56_O_7_, (HRESIMS m/z 623.3926 [M + Na]^+^), indicating nine degrees of unsaturation. The absorption at 238 nm in the UV spectrum exhibited a conjugated double bond group. The ^1^H NMR spectrum of **4** (Table 1) showed signals allocable to seven methyl groups [*δ*_H_ 1.73, 1.72, 1.46, 1.04, 0.87, 0.87, 0.77], five olefinic protons [*δ*_H_ 6.33 (1H, dd, *J* = 10.8, 15.0 Hz), 6.17 (1H, dd, *J* = 1.8, 10.2 Hz), 5.91 (1H, d, *J* = 10.2 Hz), 5.53 (1H, dd, *J* = 3.6, 9.6 Hz), 5.48 (1H, dd, *J* = 8.4, 14.4 Hz)], and a *β*-allopyranoside moiety [*δ*_H_ 5.38 (1H, d, *J* = 7.8 Hz, H-1′)] [21]. After acid hydrolysis of **4** with HCl/MeOH, d-allose was detected by TLC and specific rotation comparing with the standard. The ^13^C NMR and DEPT spectrum (Table 2) of **4** revealed signals assignable to the sugar moiety and tetracylic part were very semblable to those of (23*E*)-5*β*,19-epoxycucurbita-6,23,25-trien-3*β*-ol 3-*O*-*β*-d-allopyranoside (charantosides IV) [15], while the signals of side chain were significantly disparate. The olefinic carbons at *δ*_C_ 138.9, 124.6, 126.1, 132.1 and their coupling constants in the ^1^H-NMR spectrum [*δ*_H_ 5.48 (1H, dd, 8.4, 14.4), 6.33 (1H, dd, 10.8, 15.0), 5.91 (1H, d, 10.2)] implied that a conjugated double bond existed in the side chain. This was further confirmed via the ^1^H-^1^H COSY correlations of H-21/H-20/H-22/H-23/H-24 and the key HMBC correlations Me-21/C-17, C-20, C-22, H-22/C-21, C-24, H-24/C-22, C-23, H-26/C-24, C-25, C-27, and H-27/C-24, C-25, C-26 (Fig. 2). Therefore, the structure of the side chain was almost identical to 5*β*,19-epoxy-cucurbita-6,22*E*,24-trien-3*β*-ol [22]. Based on the above observation, compound **4** was identified as 5*β*,19-epoxy-cucurbita-6,22*E*,24-trien-3*β*-ol 3-*O*-*β*-d-allopyranoside, and named momorcharacoside A.

Compound **5** was obtained as white amorphous powder and assigned a molecular formula of C_37_H_60_O_9_, (HRESIMS m/z 671.4136 [M + Na]^+^), indicating eight degrees of unsaturation. The ^1^H NMR spectrum of **5** (Table 1) showed signals assignable to seven methyl groups [*δ*_H_ 1.54, 1.54, 1.54, 0.92, 0.89, 0.82, 0.79], one methoxy groups [*δ*_H_ 3.44], four olefinic protons [*δ*_H_ 6.28 (1H, d, *J* = 9.6 Hz), 5.92 (1H, overlap), 5.92 (1H, overlap), 5.48 (1H, dd, *J* = 3.6, 9.6 Hz)], and a *β*-glucopyanosyl moiety [*δ*_H_ 4.92 (1H, d, *J* = 7.8 Hz, H-1′)] [18, 21]. The suger moiety were determined to be d-glucose on the basis of acidic hydrolysis and TLC and specific rotation analysis. The ^13^C NMR (Table 2) showed 37 carbon signals, which were closely similar to those of 19(*R*)-methoxy-5*β*,19-epoxycucurbita-6,23-diene-3*β*,25-diol 3-*O*-*β*-d-glucopyranoside (goyaglycoside-a) [23] except for the signals due to the stereochemistry at C-19. Thus, the Δ*δ*_C_ values [*δ*_C_ (goyaglycoside-a) -−*δ*_C_ (**5**)] for the relevant signals were feckly consistent with those reported in the literature [19]. Therefore, compound **5** has the (*S*)-configuration at C-19, which was further confirmed by the ROESY correlation (Fig. 3) of H-8/H-19. Based on the above proof, compound **5** was identified as 19(*S*)-methoxy-5*β*,19-epoxycucurbita-6,23-dien-3*β*,25-diol 3-*O*-*β*-d-glucopyranoside, named goyaglycoside-l.

In this study, five new and five known compounds were isolated from *M. charantia*, all of which were cucurbitane-type triterpene glycosides. All compounds were evaluated for their *α*-glucosidase inhibitory activities with acarbose as a positive control. Compounds **2**, **5**, **7**, **8**, and **9** showed moderate inhibitory activities with IC_50_ values of 63.26 ± 3.04, 59.13 ± 4.67, 35.08 ± 4.15, 36.38 ± 3.03, 28.40 ± 2.08 μM, respectively. The IC_50_ value of positive control (acarbose) was 87.65 ± 6.51 μM (Table 3). Interestingly, all the active compounds contained *β*-d-glucopyranosyl, suggesting that the presence of glucose groups may affect the activity of triterpenes. However, further studies are needed to determine the structure–activity relationship of the cucurbitacene-type triterpenes. The results of this study also showed that cucurbitane-type triterpene glycosides might be the key ingredient in the hypoglycemic effect of *M. charantia*, some of them had significant blood sugar lowering effect.

**Table 3:** α-Glucosidase inhibitory activities of compounds **1**–**10**

Compounds	IC_50_ (*μ*M)	Compounds	IC_50_ (*μ*M)
**1**	> 100	**6**	> 100
**2**	63.26 ± 3.04	**7**	35.08 ± 4.15
**3**	> 100	**8**	36.38 ± 3.03
**4**	> 100	**9**	28.40 ± 2.08
**5**	59.13 ± 4.67	**10**	> 100
Acarbose	87.65 ± 6.51 (positive control)		

## Experimental Section

### General Experimental Procedures

UV spectra were recorded on a UV-2401PC spectrometer (Shimadzu, Kyoto, Japan). Optical rotations were measured in methanol on JASCO P-1020 digital polarimeter (Jasco, Tokyo, Japan). IR spectra were scanned on a Bruker Tensor-27 Fourier transform infrared spectrometer with KBr pellets (Bruker, German). High-resolution (HR) ESI mass spectra data were measured on a Waters API QSTAR Pulsar spectrometer. 1D and 2D NMR spectra were obtained in pyridine-*d*_5_ on Bruker Ascend-400, 600 and 800 MHz NMR spectrometers with tetramethylsilane (TMS) as internal standard (Bruker, Zurich, Switzerland). Column chromatography (CC) was performed on macroporous resin (D-101, Tianjin, China), Lichroprep RP-18 (Merck, German), sephadex LH-20 (Pharmacia, USA), silica gel (200–300 mesh, Qingdao, China), and Semi-preparative HPLC was performed on an Agilent 1260 liquid chromatography system equipped with a ZORBAX SB-C18 column (5 μm, 9.4 × 250 mm, 3.0 mL/min) and a DAD detector. Fractions were detected by TLC, and spots were visualized by spraying with 10% H_2_SO_4_ in EtOH, followed by heating. *α*-glucosidase inhibitory activity was evaluated on the basis of the ability of the compounds to decrease glucosidase activity and then inhibit the breaking of glycosidic bonds in *p*-nitrophenyl-*α*-d-glucopyranoside (PNPG). Water was purchased from wahaha group co. LTD. Acetonitrile (chromatographic grade) was purchased from OCEANPAK (Sweden). Common organic solvents are industrial grade, used after re-distillation. PNPG was obtained from Sigma Chemical Co. (St. Louis, Mo, USA). *α*-glucosidase was purchased from Shanghai yuanye biotechnology Co., Ltd. Potassium phosphate buffer solution (PPBS) was obtained from Shanghai Yidian Scientific Instrument Co., Ltd. 96-well plates was purchased from Nest biotechnology co., LTD.

### Plant Material

Dried slices of *M. charantia* were purchased from the Luosiwan Chinese Herbal Medicine Market in Kunming, Yunnan Province, China, in February 2017. The material was identified by associate Prof. Jian-Chao Cheng from Kunming Institute of Botany (KIB), Chinese Academy of Science (CAS). A specimen was deposited in the State Key Laboratory of Phytochemistry and Plant Resource in West China, Kunming Institute of Botany, Kunming, China.

### Extraction and Isolation

The dried fruits of *M. charantia* (40.0 kg) were sliced and extracted with MeOH. The solution was concentrated under reduced pressure to obtain a crude extract (25 kg), which was then successfully partitioned with petroleum ether (PE), EtOAc (EA), and n-butanol, respectively. The EtOAc fraction (2.0 kg) was subjected to the D101 macroporous resin, eluting with gradient system of MeOH/H_2_O (30:70, 50:50, 70:30, 90:10, 100:1) to afford five fractions. The fraction (MeOH/H_2_O 90:10, 87.0 g) was chromatographed on a silica gel column, eluting with gradient system of CHCl_3_/MeOH (100:1–1:1) to give four fractions (Fr.1–Fr.4). Fr.2 (16.8 g) was applied to ODS column, eluting with MeOH/H_2_O to give six sub fractions (Fr.2.1–Fr.2.6). Fr.2.5 (4.2 g) was separated over silica gel column (PE/EA) followed by semi-preparative HPLC (CH_3_CN/H_2_O), to yield compounds **1** (1.0 mg), **2** (1.0 mg), **3** (2.5 mg), and **10** (4.0 mg), respectively. Fr.2.6 (2.1 g) was successively purified by open column CC (CHCl_3_/MeOH) and semi-preparative HPLC (CH_3_CN/H_2_O), respectively, to afford compounds **4** (28.0 mg), **6** (6.0 mg), **7** (5.0 mg), **8** (1 mg), and **9** (30.0 mg). Similarly, Fr.3 (643 mg) was purified by RP-HPLC with CH_3_CN /H_2_O as eluent to obtain compounds **5** (10.0 mg).

#### Charantoside H (**1**)

White amorphous powder; [*α*]_D_^19^ − 49.62 (*c* 0.11, MeOH); UV (MeOH) λ_max_ (log ε) 196 (4.22) nm; IR (KBr) *ν*_max_ 3426, 3027, 2926, 2875, 2815, 1736, 1634, 1465, 1447, 1380, 1320, 1292, 1260, 1218, 1182, 1154, 1108, 1084, 1049, 1033, 986, 953, 926, 882, 843, 801, 775, 747, 721, 696, 557, 529, 513, 467, 440, 411, 402 cm^−1^; For ^1^H NMR and ^13^C NMR (pyridine-*d*_5_) spectroscopic data, see Tables 1 and Table 2; HRESIMS m/z 685.4295 [M + Na]^+^ (calcd for C_38_H_62_O_9_Na, 685.4286).

#### Charantoside J (**2**)

White solid; [*α*]_D_^20^ − 88.78 (*c* 0.11, MeOH); UV (MeOH) λ_max_ (log ε) 196 (4.33) nm; IR (KBr) *ν*_max_ 3424, 3026, 2928, 2875, 2815, 1736, 1636, 1465, 1446, 1383, 1320, 1292, 1260, 1218, 1183, 1153, 1110, 1086, 1050, 1033, 987, 952, 919, 817, 801, 775, 747, 721, 695, 618, 582, 557, 529, 513, 466, 411, 402 cm^−1^; For ^1^H NMR and ^13^C NMR (pyridine-*d*_5_) spectroscopic data, see Table 1 and Table 2; HRESIMS m/z 685.4293 [M + Na]^+^ (calcd for C_38_H_62_O_9_Na, 685.4286).

#### Charantoside K (**3**)

White amorphous powder; [*α*] − 35.77 (*c* 0.13, MeOH); UV (MeOH) λ_max_ (log ε) 196 (4.15) nm; IR (KBr) *ν*_23D__max_ 3428, 3028, 2925, 2874, 1736, 1630, 1465, 1448, 1377, 1307, 1287, 1260, 1212, 1197, 1180, 1155, 1108, 1082, 1049, 1033, 985, 953, 941, 926, 882, 843, 803, 778, 755, 731, 691, 550, 522, 496, 467, 449, 440, 411, 402 cm^−1^; For ^1^H NMR and ^13^C NMR (pyridine-*d*_5_) spectroscopic data, see Tables 1 and 2; HRESIMS m/z 685.4296 [M + Na]^+^ (calcd for C_38_H_62_O_9_Na, 685.4286).

#### Momorcharacoside A (**4**)

White amorphous powder; [*α*]_D_^24^ − 53.88 (*c* 0.29, MeOH); UV (MeOH) λ_max_ (log ε) 238 (4.02), 196 (4.07), 211(3.73) nm; IR (KBr) *ν*_max_ 3391, 3124, 2949, 2873, 2387, 2318, 1637, 1592, 1469, 1397, 1377, 1348, 1310, 1085, 1034, 1000, 777, 749, 684, 661, 628, 586, 531, 493, 410 cm^−1^; For ^1^H NMR and ^13^C NMR (pyridine-*d*_5_) spectroscopic data, see Tables 1 and 2; HRESIMS m/z 623.3926 [M + Na]^+^ (calcd for C_36_H_56_O_7_Na, 623.3918).

#### Goyaglycoside-l (**5**)

White amorphous powder; [*α*]_D_^20^ − 71.96 (*c* 0.14, MeOH); UV (MeOH) λ_max_ (log ε) 196 (4.21) nm; IR (KBr) *ν*_max_ 3427, 3027, 2970, 2947, 2927, 2873, 1735, 1632, 1464, 1449, 1377, 1312, 1288, 1256, 1199, 1158, 1138, 1112, 1078, 1050, 980, 950, 941, 925, 844, 803, 778, 753, 733, 695, 579, 549, 529, 491, 466, 450, 439, 429, 412 cm^−1^; For ^1^H NMR and ^13^C NMR (pyridine-*d*_5_) spectroscopic data, see Tables 1 and 2; HRESIMS m/z 671.4136 [M + Na]^+^ (calcd for C_37_H_60_O_9_Na, 671.4130).

### Acid Hydrolysis of Compounds **4** and **5** for Sugar Analysis

Compounds **4 **and** 5** (5 mg each), were separately dissolved in 2 M HCl/CH_3_OH (1:1, 5 mL) and heated at 80 °C for 4 h in a water bath. CHCl_3_/H_2_O 1:1 (5 mL × 3) was used for extraction. The aqueous phase was neutralized with Na_2_CO_3_. Each H_2_O layer was concentrated in vacuo to give a monosaccharide, which was identified by TLC [BuOH/acetic ether/H_2_O (4:1:5 upper layer)] and specific rotation compared with the authentic samples, All: *R*_f_ = 0.47, [*α*]_D_^21^ =  + 24.4; Glc: *R*_f_ = 0.51, [*α*]_D_^21^ =  + 51.9.

### *α*-Glucosidase Inhibitory Activity

The *α*-glucosidase inhibition assay was performed according to the method adapted from the literature with slight modifications [24]. *α*-glucosidase can cut glycosidic bonds in the PNPG to produce 4-nitrophenol (yellow), then measured its absorbance can determine the activity of enzyme. The test samples and ursolic acid (positive control) were dissolved in dimethylsulfoxide (DMSO), and then diluted with PPBS (pH 6.86) to the required concentration. The *α*-glucosidase (1.0 U/mL) and substrate (PNPG, 2.5 mM) were dissolved in PPBS. Sample wells included 60 *μ*L of PPBS, 10 μL of test substances, 30 μL of enzyme stock solution, and incubated at 37 °C for 10 min. After the pre-incubation phase, 40 μL of PNPG solution was added and the mixture was incubated for another 20 min at 37 °C. Finally, 80 μL Na_2_CO_3_ (0.2 M) solution was added to the sample wells to stop the reaction. The absorbance of the reaction mixture was recorded at 405 nm using a microplate reader. All samples were measured in triplicate. The inhibition rate (%) was calculated by the following formula: Inhibition (%) = [1 −  (A_sample_/A_control_)] × 100.

## Electronic supplementary material

Below is the link to the electronic supplementary material.

Supplementary file1 (DOCX 120873 kb)

## References

[1] G.H. Wu, Y.X. Zang, Y.X. Liu,* Chinese Health Management Dictionary* (2001), 468 pp

[2] IDF diabetes atlas (2019), https://www.diabetesatlas.org/. Accessed 6 Feb 2020

[3] Stuermer EK, Besser M, Terberger N, Koester V, Bachmann HS, Severing AL (2019). Naunyn-Schmiedebergs Arch. Pharmacol..

[4] Peter EL, Kasali FM, Deyno S, Mtewa A, Nagendrappa PB, Tolo CU, Ogwang PE, Sesaazi D (2019). J. Ethnopharmacol..

[5] Zhang C, Huang M, Hong R, Chen H (2019). Int. J. Biol. Macromol..

[6] Shivanagoudra SR, Perera WH, Perez JL, Athrey G, Sun YX, Jayaprakasha GK, Patil BS (2019). Bioorg. Chem..

[7] Jiang S, Xu L, Xu Y, Guo YS, Wei L, Li XT, Song W (2020). Electron. J. Biotechnol..

[8] Chhabra G, Dixit A (2013). Bioinformation.

[9] Shan B, Xie JH, Zhu JH, Peng Y (2012). Food Bioprod. Process..

[10] Jia S, Shen M, Zhang F, Xie J (2017). Int. J. Mol. Sci..

[11] Hsiao PC, Liaw CC, Hwang SY, Cheng HL, Zhang LJ, Shen CC, Hsu FL, Kuo YH (2013). J. Agric. Food Chem..

[12] Lee SY, Eom SH, Kim YK, Park NI, Park SU (2009). J. Med. Plants Res..

[13] Yue J, Sun Y, Xu J, Zhang X, Zhao Y (2020). J. Nat. Med..

[14] J. Qi, X. Cao, Y. Sun, L. Cheng, Patent No. CN107556362A (2018)

[15] Akihisa T, Higo N, Tokuda H, Ukiya M, Akazawa H, Tochigi Y, Kimura Y, Suzuki T, Nishino H (2007). J. Nat. Prod..

[16] Okabe H, Miyahara Y, Yamauchi T (1982). Tetrahedron. Lett..

[17] Liu Y, Ali Z, Khan IA (2008). Planta Med..

[18] Nakamura S, Murakami T, Nakamura J, Kobayashi H, Matsuda H, Yoshikawa M (2006). Chem. Pharm. Bull..

[19] Zeng K, He YN, Yang D, Cao JQ, Xia XC, Zhang SJ, Bi XL, Zhao YQ (2014). Eur. J. Med. Chem..

[20] Liaw CC, Huang HC, Hsiao PC, Zhang LJ, Lin ZH, Hwang SY, Hsu FL, Kuo YH (2015). Planta Med..

[21] Yue J, Sun Y, Xu J, Cao J, Chen G, Zhang H, Zhang X, Zhao Y (2019). Phytochemistry.

[22] Cao JQ, Zhang BY, Zhao YQ (2013). Chin. Herb. Med..

[23] Murakami T, Emoto A, Matsuda H, Yoshikawa M (2001). Chem. Pharm. Bull..

[24] Perez JL, Jayaprakasha GK, Patil BS (2019). Food Chem..

