# Supplementary material for: Cucurbitane-Type Triterpene Glycosides from Momordica charantia and Their α-Glucosidase Inhibitory Activities
Source: Nat Prod Bioprospect. 2020 May 6;10(3):153–61. doi: 10.1007/s13659-020-00241-5 (PMC7253571; doi:10.1007/s13659-020-00241-5)
Supplement: Supplementary file 1 — Supplementary file1 (DOCX 120873 kb) [file 13659_2020_241_MOESM1_ESM.docx]

Supporting information

**Cucurbitane-type Triterpene Glycosides with Their *α*-Glucosidase Inhibitory Activities from** ***Momordica charantia***

Ya Gao^a,b^, Jian-Chao Chen^a^, Xing-Rong Peng^a^, Zhong-Rong Li^a^, Hai-Guo Su^a^ and Ming-Hua Qiu^a,b,c *^

^a^ State Key Laboratory of Phytochemistry and Plant Resources in West China, Kunming Institute of Botany, Chinese Academy of Sciences, Kunming 650201, People’s Republic of China

^b^ University of the Chinese Academy of Sciences, Beijing 100049, People’s Republic of China

^c^ Yunnan Key Laboratory of Natural Medicinal Chemistry, Chinese Academy of Sciences, Kunming 650201, People’s Republic of China

* **Correspondence**

Prof. Ming-Hua Qiu

State Key Laboratory of Phytochemistry and Plant Resources in West China, Kunming Institute of Botany, Chinese Academy of Sciences, Kunming 650201, P. R. China

Tel: +86-871-65223327

Fax: +86-871-65223255

Email address: [mhchiu@mail.kib.ac.cn](mailto:mhchiu@mail.kib.ac.cn)

**Supporting Information List**

Characterization Data of New Compounds

**S1.** ^1^H NMR spectrum of compound **1**

**S2.** ^13^C NMR spectrum of compound **1**

**S3.** HSQC spectrum of compound **1**

**S4.** HMBC spectrum of compound **1**

**S5.** ^1^H-^1^H COSY spectrum of compound **1**

**S6.** ROESY spectrum of compound **1**

**S7.** HR-ESIMS spectrum of compound **1**

**S8.** ^1^H NMR spectrum of compound **2**

**S9.** ^13^C NMR spectrum of compound **2**

**S10.** HSQC spectrum of compound **2**

**S11.** HMBC spectrum of compound **2**

**S12.** ^1^H-^1^H COSY spectrum of compound **2**

**S13.** ROESY spectrum of compound **2**

**S14.** HR-ESIMS spectrum of compound **2**

**S15.** ^1^H NMR spectrum of compound **3**

**S16.** ^13^C NMR spectrum of compound **3**

**S17.** HSQC spectrum of compound **3**

**S18.** HMBC spectrum of compound **3**

**S19.** ^1^H-^1^H COSY spectrum of compound **3**

**S20.** ROESY spectrum of compound **3**

**S20.** HR-ESIMS spectrum of compound **3**

**S22.** ^1^H NMR spectrum of compound **4**

**S23.** ^13^C NMR spectrum of compound **4**

**S24.** HSQC spectrum of compound **4**

**S25.** HMBC spectrum of compound **4**

**S26.** ^1^H-^1^H COSY spectrum of compound **4**

**S27.** ROESY spectrum of compound **4**

**S28.** HR-ESIMS spectrum of compound **4**

**S29.** ^1^H NMR spectrum of compound **5**

**S30.** ^13^C NMR spectrum of compound **5**

**S30.** HSQC spectrum of compound **5**

**S32.** HMBC spectrum of compound **5**

**S33.** ^1^H-^1^H COSY spectrum of compound **5**

**S34.** ROESY spectrum of compound **5**

**S35.** HR-ESIMS spectrum of compound **5**

**S1.** ^1^H NMR spectrum of compound **1**

**S2.** ^13^C NMR spectrum of compound **1**

**S3.** HSQC spectrum of compound **1**

**S4.** HMBC spectrum of compound **1**

**S5.** ^1^H-^1^H COSY spectrum of compound **1**

**S6.** ROESY spectrum of compound **1**

**S7.** HR-ESIMS spectrum of compound **1**

**S8.** ^1^H NMR spectrum of compound **2**

**S9.** ^13^C NMR spectrum of compound **2**

**S10.** HSQC spectrum of compound **2**

**S11.** HMBC spectrum of compound **2**

**S12.** ^1^H-^1^H COSY spectrum of compound **2**

**S13.** ROESY spectrum of compound **2**

**S14.** HR-ESIMS spectrum of compound **2**

**S15.** ^1^H NMR spectrum of compound **3**

**S16.** ^13^C NMR spectrum of compound **3**

**S17.** HSQC spectrum of compound **3**

**S18.** HMBC spectrum of compound **3**

**S19.** ^1^H-^1^H COSY spectrum of compound **3**

**S20.** ROESY spectrum of compound **3**

**S20.** HR-ESIMS spectrum of compound **3**

**S22.** ^1^H NMR spectrum of compound **4**

**S23.** ^13^C NMR spectrum of compound **4**

**S24.** HSQC spectrum of compound **4**

**S25.** HMBC spectrum of compound **4**

**S26.** ^1^H-^1^H COSY spectrum of compound **4**

**S27.** ROESY spectrum of compound **4**

**S28.** HR-ESIMS spectrum of compound **4**

**S29.** ^1^H NMR spectrum of compound **5**

**S30.** ^13^C NMR spectrum of compound **5**

**S30.** HSQC spectrum of compound **5**

**S32.** HMBC spectrum of compound **5**

**S33.** ^1^H-^1^H COSY spectrum of compound **5**

**S34.** ROESY spectrum of compound **5**

**S35.** HR-ESIMS spectrum of compound **5**

s
